# Ectopic Expression of a R2R3-MYB Transcription Factor Gene *LjaMYB12* from *Lonicera japonica* Increases Flavonoid Accumulation in *Arabidopsis thaliana*

**DOI:** 10.3390/ijms20184494

**Published:** 2019-09-11

**Authors:** Xiwu Qi, Hailing Fang, Zequn Chen, Zhiqi Liu, Xu Yu, Chengyuan Liang

**Affiliations:** 1 Institute of Botany, Jiangsu Province and Chinese Academy of Sciences (Nanjing Botanical Garden Mem. Sun Yat-Sen), Nanjing 210014, China; qixiwu@126.com (X.Q.); fanghailing2013@163.com (H.F.); zequnchen@126.com (Z.C.); yuxu84@163.com (X.Y.); 2 Nanjing Foreign Language School, Nanjing 210008, China; lzq030729@gmail.com; 3 Division of Plant Sciences, University of Missouri, Columbia, MO 65211-7145, USA; 4 Jiangsu Key Laboratory for the Research and Utilization of Plant Resources, Nanjing 210014, China

**Keywords:** *Lonicera japonica* Thunb., *LjaMYB12*, transcription regulation, flavonoid biosynthesis

## Abstract

*Lonicera japonica* Thunb. is a widely used medicinal plant and is rich in a variety of active ingredients. Flavonoids are one of the important components in *L. japonica* and their content is an important indicator for evaluating the quality of this herb. To study the regulation of flavonoid biosynthesis in *L. japonica*, an R2R3-MYB transcription factor gene *LjaMYB12* was isolated and characterized. Bioinformatics analysis indicated that *LjaMYB12* belonged to the subgroup 7, with a typical R2R3 DNA-binding domain and conserved subgroup 7 motifs. The transcriptional level of *LjaMYB12* was proportional to the total flavonoid content during the development of *L. japonica* flowers. Subcellular localization analysis revealed that LjaMYB12 localized to the nucleus. Transactivation activity assay indicated that LjaMYB12 was a transcriptional activator. Then, ectopic expression of *LjaMYB12* in Arabidopsis could increase PAL activity and flavonoid content and promote transcription of a range of flavonoid biosynthetic genes. Interestingly, the fold changes of downstream genes in the flavonoid biosynthetic pathway were significantly higher than that of the upstream genes, which suggested that *LjaMYB12* may have different regulatory patterns for the upstream and downstream pathways of flavonoid biosynthesis. The results provided here will effectively facilitate the study of subgroup 7 MYBs and transcriptional regulation of flavonoid biosynthesis in *L. japonica*.

## 1. Introduction

*Lonicera japonica* Thunb. is an important medicinal plant that has a wide range of applications in traditional Chinese medicine [1]. Modern pharmacological studies have revealed that *L. japonica* and its extracts have a variety of biological and pharmacological properties, such as antioxidant, anti-inflammatory, antiviral, anti-tumor and so on [2,3,4,5]. *L. japonica* is rich in a lot of active ingredients and more than 140 compounds have been isolated and identified from it [1], including phenolic acids, flavonoids, essential oils, triterpenoid saponins, and so on [6,7,8,9]. Among them, flavonoids are a major class of active ingredients and the content of luteoloside is typically used as an important indicator of quality for evaluating *L. japonica* [10].

Flavonoids are a diverse group of polyphenolic secondary metabolites, which play important roles in plant development and defense [11]. According to the difference in structure, flavonoids can be divided into different groups, including flavanones, flavones, flavonols, proanthocyanidins, anthocyanins, isoflavonoids, and so forth. The biosynthesis of flavonoids has been extensively studied, which is derived from the general phenylpropanoid pathway and then catalyzed by a series of enzymes [12,13]. This pathway has been well-characterized in model plant *Arabidopsis thaliana* and most genes encoding flavonoid biosynthetic enzymes have been isolated and characterized [14]. It is also known as a well model for the study of transcriptional regulation. The expression levels of flavonoid biosynthetic genes are regulated by a lot of transcription factors including MYB transcription factors, basic helix-loop-helix (bHLH), and WDR factors [15,16,17]. Among them, MYB proteins have been demonstrated as key factors for the regulation of flavonoid biosynthesis [18].

The MYB transcription factor family is large and functionally diverse in all eukaryotes [19,20]. It is characterized by a highly conserved N-terminal DNA-binding domain known as MYB domain. According to the number of adjacent MYB domains, MYB transcription factors can be divided into four different classes and most plant MYB genes encode proteins of the R2R3-MYB class [21,22,23,24]. In Arabidopsis, a total of 126 R2R3-MYBs have been identified, which can be divided into some subgroups based on the conservation of the DNA binding domain and amino acid motifs in the C terminus [22]. MYB transcription factors regulating the flavonoid biosynthesis have been widely investigated in Arabidopsis. For instance, *AtMYB75*, *AtMYB90*, *AtMYB113*, and *AtMYB114* (subgroup 6) could control anthocyanin biosynthesis [25] and *AtMYB123* (subgroup 2) could control proanthocyanidins biosynthesis [26].

Among the flavonoid biosynthesis related MYBs, AtMYB11, AtMYB12, and AtMYB111 (subgroup 7) have been considered as flavonol-specific regulator in Arabidospis, which could increase flavonol accumalation by up-regulating expression levels of biosynthetic genes [27,28]. Some homologous genes of other plants exhibit similar regulation function. For example, overexpression of *CcMYB12* from *Cynara cardunculus var. scolymus* in Arabidopsis can activate the expression of endogenous flavonol biosynthesis genes, leading to an increase of flavonol accumulation [29]. However, previous studies have indicated that transcription factors may have different specificities for target genes in different plants. In tomato, fruit-specific expression of *AtMYB12* can activate both the flavonol biosynthetic pathway and the caffeoyl quinic acid biosynthetic pathway [30]. Co-expression of *AtMYB12* and soybean *GmIFS1* in tobacco leads to enhanced biosynthesis of both flavonols and isoflavones [31].

In this study, we reported the isolation and functional characterization of an R2R3-MYB transcription factor gene *LjaMYB12* in *L. japonica*. The transcriptional level of *LjaMYB12* was consistent with the total flavonoid content during the development of *L. japonica* flowers. Then, we conducted subcellular localization assay, transactivation analysis, and ectopic expression in Arabidopsis to clarify the regulation function of *LjaMYB12*.

## 2. Results

### 2.1. LjaMYB12 Is a R2R3-MYB Transcription Factor in L. japonica

**Figure 1:**
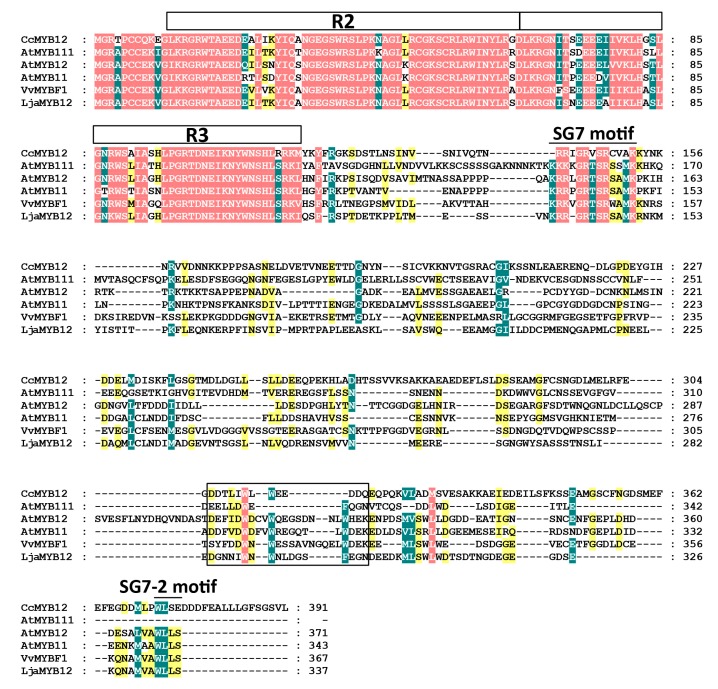
Multiple sequence alignment of LjaMYB12 and other subgroup 7 R2R3-MYBs. The position of the R2R3-MYB domain, SG7 motif and SG7-2 motif is indicated above the alignment and the predicted activation domain is highlighted by black box. Red, green, and yellow shades indicate sequence conserved percentage of 100%, 80%, and 60%, respectively. Proteins used for alignment are AtMYB11, AtMYB12 and AtMYB111 from *A. thaliana*, VvMYBF1 (ACT88298.1) from *Vitis vinifera*, CcMYB12 (AXF92691.1) from *C. cardunculus var. scolymus*, and LjaMYB12 from *L. japonica*.

We isolated an *AtMYB12* homologous gene from *L. japonica* and designated as *LjaMYB12* (GenBank accession number: MN231639). Sequence analysis indicated that the coding sequence of *LjaMYB12* was 1014 bp and could encode 337 amino acid residues. The molecular weight (Mw) and isoelectric point (pI) of the protein were 37.97 kDa and 5.05, respectively. Domain prediction showed that LjaMYB12 was a typical R2R3-MYB protein with two MYB repeats at the amino terminus. Multiple sequence alignment showed that LjaMYB12 shared conserved R2R3 domain and subgroup 7 motifs with other subgroup 7 R2R3 transcription factors (Figure 1). The predicted activation domain of LjaMYB12 shared some conserved features with other R2R3-MYB flavonol regulators and contained several acidic amino acids (Figure 1). Phylogenetic analysis of LjaMYB12, Arabidopsis MYB family and other subgroup 7 R2R3 MYBs indicated that LjaMYB12 was clustered into one clade with other subgroup 7 MYB members (Figure S1).

### 2.2. The Transcriptional Level of LjaMYB12 Is Proportional to the Total Flavonoid Content during the Development of L. Japonica Flowers

**Figure 2:**
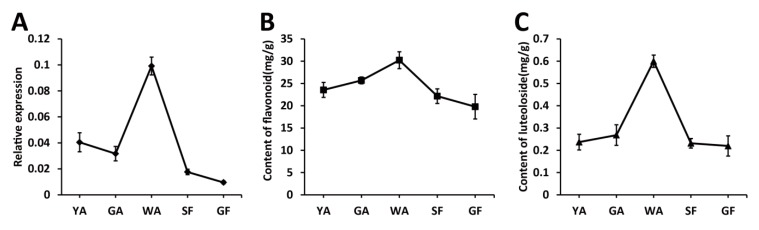
Transcriptional levels of *LjaMYB12* and flavonoid contents in flower buds of *L. japonica* at different developmental stages. (**A**). Transcriptional patterns of *LjaMYB12*. (**B**). Flavonoid contents. (**C**). Luteoloside contents. YA-young alabastrum, GA-green alabastrum, WA-white alabastrum, SF-silvery flower, and GF-golden flower.

Flower bud is the main medicinal part of *L. japonica*, which accumulates a lot of active ingredients including flavonoids. In this study, flower buds in five different developmental stages were selected to explore the relationship of *LjaMYB12* expression and flavonoid contents. qRT-PCR results indicated that the expression levels of *LjaMYB12* increased first and then decreased, reaching the highest level in the while alabastrum stage (Figure 2A). Then, the contents of flavonoids and luteoloside in flower buds at different developmental stages were measured. The results revealed that the contents of flavonoids and luteoloside increased first and then decreased with the development of flower buds, reaching the highest level in the while alabastrum stage (Figure 2B,C). Comparing the results of gene expression and ingredient quantification, we found that the transcriptional level of *LjaMYB12* is proportional to the total flavonoid and luteoloside contents during the development of *L. japonica* flowers.

### 2.3. LjaMYB12 Localizes to the Nucleus

**Figure 3:**
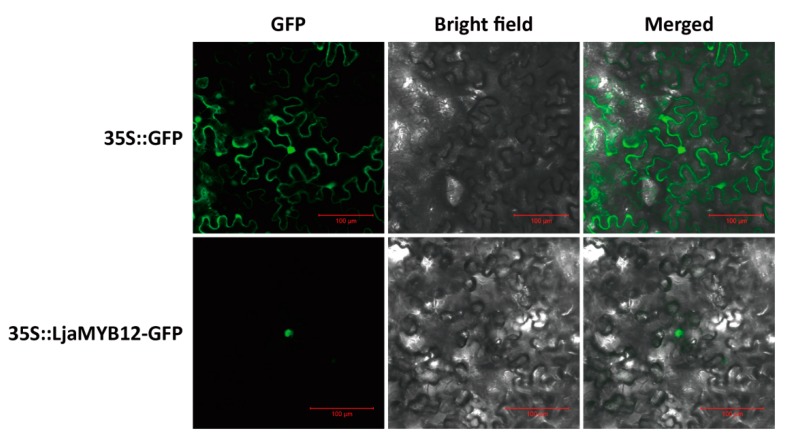
Subcellular localization of the LjaMYB12 protein in tobacco epidermal cells.

Transcription factors mainly exert their regulatory functions in the nucleus and the prediction of nuclear localization signal of LjaMYB12 suggested that it might be localized to the nucleus. To determine the subcellular localization of LjaMYB12, the ORF of *LjaMYB12* was fused to the N-terminus of GFP and the recombinant protein was transient expressed in tobacco cells by *Agrobacterium*-mediated transformation. Results showed that LjaMYB12-GFP fusion protein localized in the nucleus, whereas GFP alone presented throughout the whole cell (Figure 3).

### 2.4. LjaMYB12 Has Transactivation Activity

**Figure 4:**
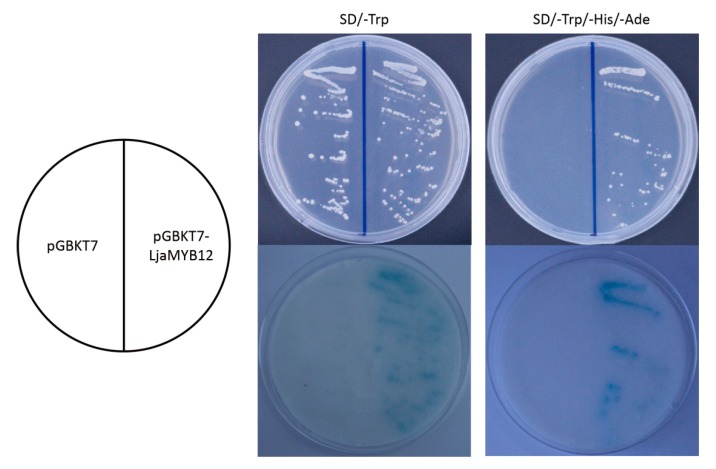
Transactivation assay of the LjaMYB12 in a yeast expression system. The transformed yeast cells were cultured on SD/-Trp or SD/-Trp/-His/-Ade medium plus 3-AT. LacZ activity was assayed using a colony-lift filter assay with β-galactosidase.

The transactivation activity of LjaMYB12 was analyzed using a yeast assay. The recombinant plasmid containing *LjaMYB12* and *GAL4* DNA binding domain was transformed into the yeast strain AH109. The transformed yeast cells were selected on SD/-Trp medium and then cultured on SD/-Trp or SD/-Trp/-His/-Ade plus 3-AT. Results revealed that both the fusion plasmid and control plasmid could survive on SD/-Trp medium but only the fusion plasmid could survive on SD/-Trp/-His/-Ade medium (Figure 4). These results indicated that the fusion effector was able to activate the expression of the *His* reporter gene. Colony-lift filter assay indicated that the expression of *LacZ* was activated by pGBKT7-LjaMYB12 fusion plasmid (Figure 4). These results indicated that LjaMYB12 was a transcriptional activator.

### 2.5. LjaMYB12 Overexpression Increases PAL Activity and Flavonoid Content in Transgenic Arabidopsis

**Figure 5:**
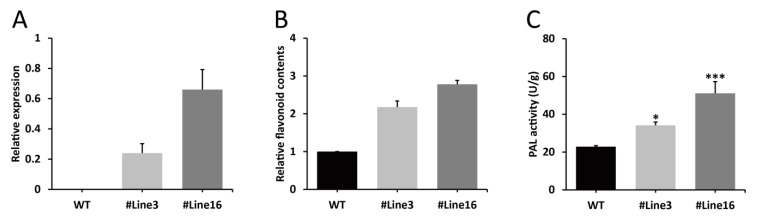
Transcriptional levels of *LjaMYB12*, flavonoid contents and PAL activities in WT and transgenic Arabidopsis. (**A**). Transcriptional levels of *LjaMYB12* in WT and transgenic Arabidopsis. (**B**). Flavonoid contents in WT and transgenic Arabidopsis. (**C**). PAL activities in WT and transgenic Arabidopsis. * and *** represent *p* < 0.05 and *p* < 0.001, respectively.

To investigate the function of *LjaMYB12* in flavonoid accumulation, overexpression of *LjaMYB12* in transgenic Arabidopsis was performed. After screening for transgenic plants by kanamycin and GUS staining, the transcriptional levels of *LjaMYB12* in Arabidopsis were detected by qRT-PCR with gene specific primers. The results showed that *LjaMYB12* was specifically expressed in transgenic plants (Figure 5A). Then, the total flavonoid contents of WT and transgenic Arabidopsis were extracted and compared. The results indicated that the content of total flavonoid in transgenic plants was significantly higher than that in WT, with fold changes of 2.18 and 2.78 for transgenic line 3 and line 16, respectively (Figure 5B). We also measured the enzyme activity of PAL in WT and transgenic Arabidopsis, which is one of the most important rate-limiting enzymes in the biosynthetic pathway of flavonoids. The results revealed that the PAL activities of transgenic line 3 and line 16 were 1.49 and 2.23 times higher than that of WT, respectively (Figure 5C).

### 2.6. LjaMYB12 Promotes Transcription of a Range of Flavonoid Biosynthetic Genes in Transgenic Arabidopsis

**Figure 6:**
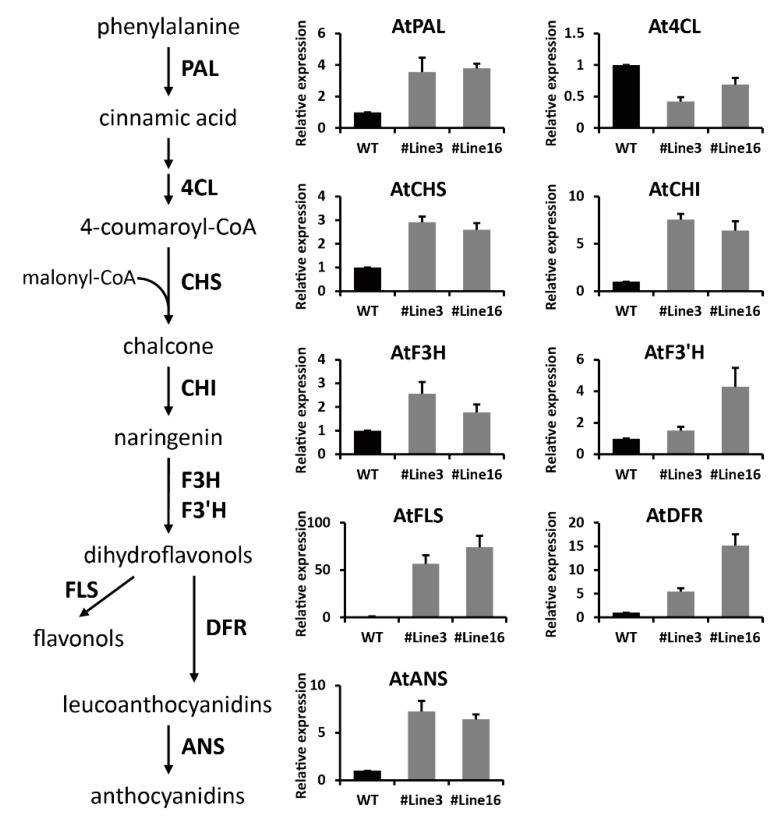
Transcriptional levels of flavonoid biosynthetic genes in WT and transgenic Arabidopsis. PAL, phenylalanine ammonia-lyase; 4CL, 4-coumarate:CoA ligase; CHS, chalcone sy nthase; CHI, chalcone isomerase; F3H, flavanone 3-hydroxylase; F3′H, flavonoid 3′-hydroxylase; FLS, flavonol synthase; DFR, dihydroflavonol 4-reductase; ANS, anthocyanidin synthase.

The transcriptional levels of nine flavonoid biosynthetic genes in WT and transgenic Arabidopsis were analyzed by qRT-PCR. The results showed that except for *At4CL*, the expression levels of the other eight genes were up-regulated in transgenic plants (Figure 6). Interestingly, the fold changes of downstream genes (*AtFLS*, *AtDFR*, and *AtANS*) in the flavonoid biosynthetic pathway were significantly higher than that of upstream genes (*AtPAL*, *AtCHS*, *AtCHI*, *AtF3H*, and *AtF3′H*). Among the nine genes, *AtFLS* exhibited the largest fold changes in expression, which were up-regulated 57-fold and 74-fold in transgenic line3 and line16, respectively (Figure 6).

## 3. Discussion

In this study, we isolated an R2R3-MYB transcription factor encoding gene *LjaMYB12* from *L. japonica*. Phylogenetic analysis and conserved motif analysis indicated that LjaMYB12 belonged to subgroup 7 MYBs, which were considered as flavonol-specific regulator in plants. The transcriptional level of *LjaMYB12* was proportional to the total flavonoid content during the development of *L. japonica* flowers. Then, subcellular localization assay indicated that LjaMYB12 was localized in the nucleus and transactivation analysis showed that LjaMYB12 was a transcriptional activator. Overexpression of *LjaMYB12* in transgenic Arabidopsis could increase the PAL activity and flavonoid content and up-regulate the transcriptional levels of flavonoid biosynthetic genes. The results provided here will effectively facilitate the study of subgroup 7 MYBs and transcriptional regulation of flavonoid biosynthesis in *L. japonica*.

Because the methodology of genetically modified *L. japonica* is not mature as yet, we ectopically express *LjaMYB12* in Arabidopsis to study its regulation on flavonoid biosynthesis. In *LjaMYB12*-expressing Arabidopsis, the transcriptional levels of most flavonoid biosynthetic genes were up-regulated except for *At4CL*, which showed 0.42-fold and 0.68-fold in transgenic line3 and line16, respectively. 4CL could generate 4-coumaroyl CoA and caffeoyl CoA from their respective acids, which participated in the biosynthesis of lignin, flavonoids, and hydroxycinnamoyl esters. Li et al. identified four isoforms of *4CL* in Arabidopsis and their results indicated that the four isoforms of *4CL* have overlapping yet distinct roles in phenylpropanoid metabolism [32]. In our study, the down-regulation of *At4CL* we tested might be compensated by other isoforms of *4CL*. According to the conservation of the N-terminal MYB domain and C-terminal motifs, R2R3-MYB transcription factors could be divided into many subgroups [22]. Members of the same subgroup usually exhibit similar regulatory functions. For example, the subgroup 7 of Arabidopsis R2R3-MYBs contains *AtMYB11*, *AtMYB12*, and *AtMYB111*, all of which specifically regulate flavonol biosynthesis [27,28]. *AtMYB75*, *AtMYB90*, *AtMYB113*, and *AtMYB114* belong to subgroup 6, which are involved in the regulation of anthocyanin biosynthesis [25]. *AtMYB52*, *AtMYB54*, and *AtMYB69* belong to subgroup 21 and are proposed to regulate cell wall thickening in fiber cells [33]. In different plants, the functions of same subgroup MYB proteins are broadly conserved. Previous studies have shown that *ROSEA1* and *ROSEA2* in snapdragon, *MdMYB10* in apple, *AN2* in petunia, and *FaMYB10* in strawberry belong to subgroup 6, which have similar function as the Arabidopsis subgroup 6 to regulate anthocyanin biosynthesis [34,35,36,37,38]. *GtMYBP3* and *GtMYBP4* in Japanese gentian, *VvMYBF1* in grape and *CcMYB12* in *C. cardunculus var. scolymus* belong to subgroup 7, which also have similar function as the Arabidopsis subgroup 7 to regulate flavonol biosynthesis [29,39,40]. In this study, the expression levels of flavonol biosynthetic genes including *AtCHS*, *AtCHI*, *AtF3H* and *AtFLS* were significantly up-regulated by the ectopic expression of *LjaMYB12* in Arabidopsis, especially the flavonol-specific gene, *AtFLS*, which exhibited the largest fold changes in expression. These results indicate that *LjaMYB12* has similar function in regulation of flavonol biosynthesis with other subgroup 7 homologs.

An interesting phenomenon is that transcription factors may have different specificities for target genes in different plants. For example, in Arabidopsis, *AtMYB12* participated in the activation of genes encoding CHS, CHI, F3H, and FLS, which together determine flavonol content [27]. But ectopic expression of *AtMYB12* in tomato led to induction of almost all of the genes involved in the biosynthesis of flavonol, CGA and their derivatives, including *PAL*, *C4H*, *4CL*, *CHS*, *CHI*, *F3H*, *F3′H*, *FLS*, *ANS*, *RT*, *GT*, *C3H*, *HCT*, and *HQT*. The induced fold-changes of target genes were quite different, genes encoding PAL, CHS, and GT exhibited more than 100-fold induction while genes encoding F3′H, C4H, 4CL, and HQT exhibited only 3–10 fold induction [30]. Ectopic expression of *GtMYBP3* and *GtMYBP4* from gentian in transgenic Arabidopsis and tobacco also led to different results. In Arabidopsis, overexpression of *GtMYBP3* and *GtMYBP4* activated the expression of flavonol biosynthetic genes and increased the flavonol accumulation. The target genes of *GtMYBP3* and *GtMYBP4* were very similar, mainly including *AtCHS*, *AtCHI*, *AtF3H*, *AtF3′H*, and *AtFLS*. However, overexpression of *GtMYBP3* and *GtMYBP4* in transgenic tobaccos led to quite different results. In *GtMYBP4*-expressing tobacco, the expression of *NtCHS*, *NtCHI*, *NtF3′H*, and *NtFLS* was up-regulated and *NtF3H* was down-regulated, which led to the increase of flavonols and decrease of anthocyanins. But in *GtMYBP3*-expressing tobacco, no significant changes in flavonoid biosynthetic gene expression and flavonol contents were observed [39]. In our study, ectopic expression of *LjaMYB12* in Arabidopsis enhanced the expression of genes involved in both flavonol biosynthesis and anthocyanin biosynthesis, including *AtPAL*, *AtCHS*, *AtCHI*, *AtF3H*, *AtF3′H*, *AtFLS*, *AtDFR*, and *AtANS*. Interestingly, the fold changes of downstream genes in the flavonoid biosynthetic pathway were significantly higher than that of upstream genes and *AtFLS* exhibited the largest fold changes. These results suggest that *LjaMYB12* may have different regulatory patterns for the upstream and downstream pathways of Arabidopsis flavonoid biosynthesis.

The most valuable flavonoid of *L. japonica* is luteoloside, which is a glycosylated flavone compound. In model plant Arabidopsis, various flavonoids, including flavonols, flavanols, and anthocyanins, have been identified, and the biosynthesis pathways have been well illustrated, but the branch of flavone biosynthesis is still unclear [41]. Therefore, the studies on biosynthesis and regulation mechanisms of luteoloside are relatively few. Although the *LjaMYB12* transcript and the luteoloside content consistently showed similar developmental trends in *L. japonica*, whether *LjaMYB12* is involved in the regulation of luteoloside is still needed for further study.

## 4. Materials and Methods

### 4.1. RNA Isolation and cDNA Synthesis of L. japonica

The *L. japonica* plants used for this study were cultured in the Germplasm Nursery in Institute of Botany, Jiangsu Province and Chinese Academy of Sciences (Nanjing Botanical Garden Mem. Sun Yat-Sen), Nanjing, Jiangsu Province. Flower bud and flower samples of five stages (young alabastrum, YA; green alabastrum, GA; while alabastrum, WA; silvery flower, SF; and golden flower, GF) were collected as our previous study [42]. For RNA isolation, approximately 0.1 g of tissue was ground with liquid nitrogen and extracted using RNAiso Plus (Takara, Dalian, China) according to the manufacturer’s instructions. After quality and concentration measurement, 3 μg of total RNA was used to synthesize the first-strand cDNA with M-MLV reverse transcriptase (Promega, Madison, WI, USA).

### 4.2. Isolation and Characterization of LjaMYB12

The candidate *LjaMYB12* gene was identified from *L. japonica* transcriptome data (NCBI SRA accession: SRP132670) and isolated by PCR amplification. The forward and reverse primers used for PCR amplification were listed in Table 1. The amplified fragments were purified and then cloned into the pMD19-T simple vector (Takara, Dalian, China). The positive clones were screened and sequenced for confirmation. For sequence analysis of *LjaMYB12*, BLAST program was performed against the NCBI database. Sequence characteristics of the deduced amino acid sequence of *LjaMYB12* were analyzed in ExPASy (https://www.expasy.org/). Multiple sequence alignment was performed using Muscle3.6 (v3.6, http://www.drive5.com/muscle/) [43]. Phylogenetic analysis was conducted using MEGA4 (v4, https://www.megasoftware.net/mega4/) with the Neighbor-Joining algorithm and bootstrap testing with 1000 replications [44]. The Newick format file of bootstrap consensus tree was exported and then modified using EvolView (v2, https://www.evolgenius.info//evolview/#login) [45].

### 4.3. Subcellular Localization Assay

The coding sequences of *LjaMYB12* (without stop codon) were fused to the N-terminus of GFP under the control of CaMV 35S promoter in the p35SGK vector. Primers used for vector construction were listed in Table 1. The recombinant vector p35SGK-LjaMYB12-GFP was introduced into *Agrobacterium tumefaciens* EHA105 by chemical transformation. Transient expression of GFP fusion protein in tobacco leaves was conducted through *Agrobacterium*-mediated transformation as described previously [46]. Localization of the fluorescence signals was observed using a laser confocal microscope (Zeiss, Oberkochen, Germany).

### 4.4. Transactivation Activity of LjaMYB12 in Yeast

The transactivation activity of LjaMYB12 was assayed in yeast cells. The full-length coding sequences were cloned into the DNA-BD vector pGBKT7 (Clontech). Primers used for vector construction were listed in Table 1. The recombinant plasmid pGBKT7-LjaMYB12 was transformed into the yeast strain AH109 harboring the *LacZ* and *HIS3* reporter genes. The transformed yeast cells were selected on SD/-Trp medium and then cultured on SD/-Trp or SD/-Trp/-His/-Ade plus 3-AT. *LacZ* activity was assayed using a colony-lift filter assay with β-galactosidase.

### 4.5. Arabidopsis Transformation

The coding sequences of *LjaMYB12* were inserted into the BamHI and SalI restriction sites of p35SGK under the control of CaMV 35S promoter and nos terminator. Primers used for vector construction were listed in Table 1. The recombinant vector p35SGK-LjaMYB12 was introduced into *A. tumefaciens* EHA105 by chemical transformation. Briefly, 0.1 μg of recombinant palsmid was added to 100 μL of *Agrobacterium* competent cells, and then successively placed on ice for 5 min, 5 min in liquid nitrogen, 5 min at 37 °C, and 5 min on ice. Then, 700 μL of the YEB liquid medium was added to the tube and shaken at 200 rpm at 28 °C for 2 h. After centrifugation, the resuspended *Agrobacterium* was cultivated in YEB medium with 20 mg/L rifampin and 50 mg/L of kanamycin at 28 °C. Then, *Agrobacterium*-mediated floral dip method was used to transform *A. thaliana Columbia-0* [47]. The plants were grown in an artificial climate chamber at 22 °C with 16 h light/8 h dark cycles. The seeds of the transgenic Arabidopsis were screened in 1/2 MS medium containing 50 mg/L kanamycin. T1 and T2 transgenic seedlings were screened and verified by RT-PCR using *LjaMYB12* primers. Positive T3 transformants were used for further analysis.

### 4.6. PAL Activity Assay

The enzyme activity of phenylalanine ammonia-lyase (PAL, EC 4.3.1.5) was measured using PAL measurement kit (Jiancheng, Nanjing, China). Briefly, the Arabidopsis leaves and extraction buffer were mixed in a ratio of 1:9 and then homogenized in ice water. After centrifugation at 10000 rpm for 10 min, 40 μL of supernatant was added to 1480 μL of reaction buffer and 400 μL of substrate to initiate the reaction. The reaction was kept at 30 °C for 30 min, then, 80 μL of termination buffer was added to terminate the reaction. The A290 of the reaction was measured by the ultraviolet spectrophotometer (MAPADA, Shanghai, China). The enzyme activity unit of PAL is defined as an increase of 0.1 in the·OD_290_ min^−1^·mL^−1^·g^−1^. All results were representative of three independent experiments.

### 4.7. Quantification of Flavonoid and Luteoloside

The extraction and quantification of flavonoid were carried out according to previous studies with minor modification [48]. Briefly, 0.5 g of dried powder was added to 10 mL of 65% ethanol and then extracted with ultrasonic wave at 50 °C for 30 min. After centrifuged at 10,000× *g* for 10 min, the supernatant was collected and the absorbance was measured with an ultraviolet spectrophotometer (MAPADA, Shanghai, China) at 340 nm. The quantification of luteoloside was conducted using Dionex U3000 HPLC system (Dionex, Sunnyvale, CA, USA) with a Welch LP-C18 column (5 μm, 150 mm × 4.6 mm) at 25 °C. The flow rate was 1 mL/min and the mobile phase consisted of acetonitrile (eluent A) and water: acetic acid (999:1, *v*/*v*, eluent B). The elution program was 0–5 min, 8–10% A; 5–25 min, 10–20% A; 25–45 min, 20–30% A; 45–55 min, 30–100% A. 10 μL of the extracted sample was injected and fractions were monitored at 350 nm. Commercial luteoloside was used as the standard to identify and quantify the luteoloside contents in all samples. All results were representative of three independent experiments.

### 4.8. Quantitative Real-Time Reverse Transcriptional PCR (qRT-PCR)

For qRT-PCR, SYBR® Premix ExTaqTM II (Takara, Dalian, China) was used to prepare reactions according to the manufacturer’s instructions. The qRT-PCR reactions were carried out using the qTOWER2.2 Real-Time PCR Systems (Analytik, Jena, Germany). The PCR program was set as follows: 94 °C for 1 min, followed by 40 cycles of 94 °C for 30 s, 60 °C for 30 s, and 72 °C for 30 s. The *L. japonica* actin gene and Arabidopsis *AtACT2* gene were used as controls to normalize the relative expression levels of target genes [40,42]. The gene relative expression levels were calculated using 2^−ΔΔ*C*t^ method [49]. Gene-specific primers used for qRT-PCR were listed in Table 1. Three independent biological replicates were conducted for each sample.

## Figures and Tables

**Table 1:** Primer sequences used in this study.

Genes	Forward Primer (5′–3′)	Reverse Primer (5′–3′)
**Gene Cloning**		
*LjaMYB12*	ATGGGGAGAGCACCATGCT	CTAAGAGAGCAGCCATGCAAC
**Subcellular Localization Assay**		
*LjaMYB12*	*GG*GGTACCATGGGGAGAGCACCATGCT	*CG*GGATCCAGAGAGCAGCCATGCAACC
*EGFP*	*CG*GGATCCATGGTGAGCAAGGGCGAG	*ACGC*GTCGACTTACTTGTACAGCTCGTCATGC
**Transactivation Analysis**		
*LjaMYB12*	*GGAATTC*CATATGATGGTGAGCAAGGGCGAG	*ACGC*GTCGACCTAAGAGAGCAGCCATGCAAC
**Arabidopsis Transformation**		
*LjaMYB12*	*CG*GGATCCATGGGGAGAGCACCATGCT	*ACGC*GTCGACCTAAGAGAGCAGCCATGCAAC
**qRT-PCR**		
*LjaMYB12*	CTTTGAAGGGAATGATGAGG	TTGCTTCTCGCTGTCTCC
*LjActin*	TGCTGGATTCTGGTGATGGT	ATTTCCCGCTCTGCTGTG
*AtPAL*	ATCGAAGTGATCCGTTACGC	ACTCCGATTGGTGTTCCTTG
*At4CL*	CGCAAACCCTTTCTTCACTC	ACTCCGTCGTCGTTTTGAAG
*AtCHS*	TGAGAACCATGTGCTTCAGG	CAGATGCATGTGACGTTTCC
*AtCHI*	TTTGTACCGTCCGTCAAGTC	CAATGACGGTGAAGATCACG
*AtF3H*	TCAGATCGTTGAGGCTTGTG	ATGTCGAAACGGAGCTTGTC
*AtF3′H*	CCTCCACCTCCGACTAGGGT	TGCTCGGCCACGGATTTA
*AtFLS*	CAACATTCCGAGGTCCAACG	TCTTCGTCGGGATCGCTTAG
*AtDFR*	GTCGGTCCATTCATCACAAC	TGAGCGTTGCATAAGTCGTC
*AtANS*	TCAAGAAAGCCGGAGAAGAG	TTGTCCACTCGCGTTGTTAG
*AtACT2*	ACCCGATGGGCAAGTCATC	CGAGGGCTGGAACAAGACTTC

## Supplementary Materials

The following are available online at https://www.mdpi.com/1422-0067/20/18/4494/s1.

## Abbreviations

bHLH	basic Helix-Loop-Helix
WDR	WD Repeats
qRT-PCR	quantitative real-time Reverse Transcriptional PCR
HPLC	High Performance Liquid Chromatography
ORF	Open Reading Frame
GFP	Green Fluorescent Protein
PAL	Phenylalanine Ammonia-Lyase
4CL	4-Coumarate:CoA Ligase
C4H	Cinnamate 4-Hydroxylase
CHS	Chalcone Synthase
CHI	Chalcone Isomerase
F3H	Flavanone 3-Hydroxylase
F3′H	Flavonoid 3′-Hydroxylase
FLS	Flavonol Synthase
DFR	Dihydroflavonol 4-Reductase
ANS	Anthocyanidin Synthase
RT	Flavonol-3-glucosiderhamnosyltransferase
GT	Flavonol-3-glucosyltransferase;
C3H	*p*-Coumaroyl ester 3-Hydroxylase
HCT	Cinnamoyl CoA shikimate/quinate Transferase
HQT	Hydroxycinnamoyl CoA Quinate Transferase.
