# Supplementary material for: Ectopic Expression of a R2R3-MYB Transcription Factor Gene LjaMYB12 from Lonicera japonica Increases Flavonoid Accumulation in Arabidopsis thaliana
Source: Int J Mol Sci. 2019 Sep 11;20(18):4494. doi: 10.3390/ijms20184494 (PMC6770605; doi:10.3390/ijms20184494)
Supplement: Supplementary file 1 [file ijms-20-04494-s001.pdf]

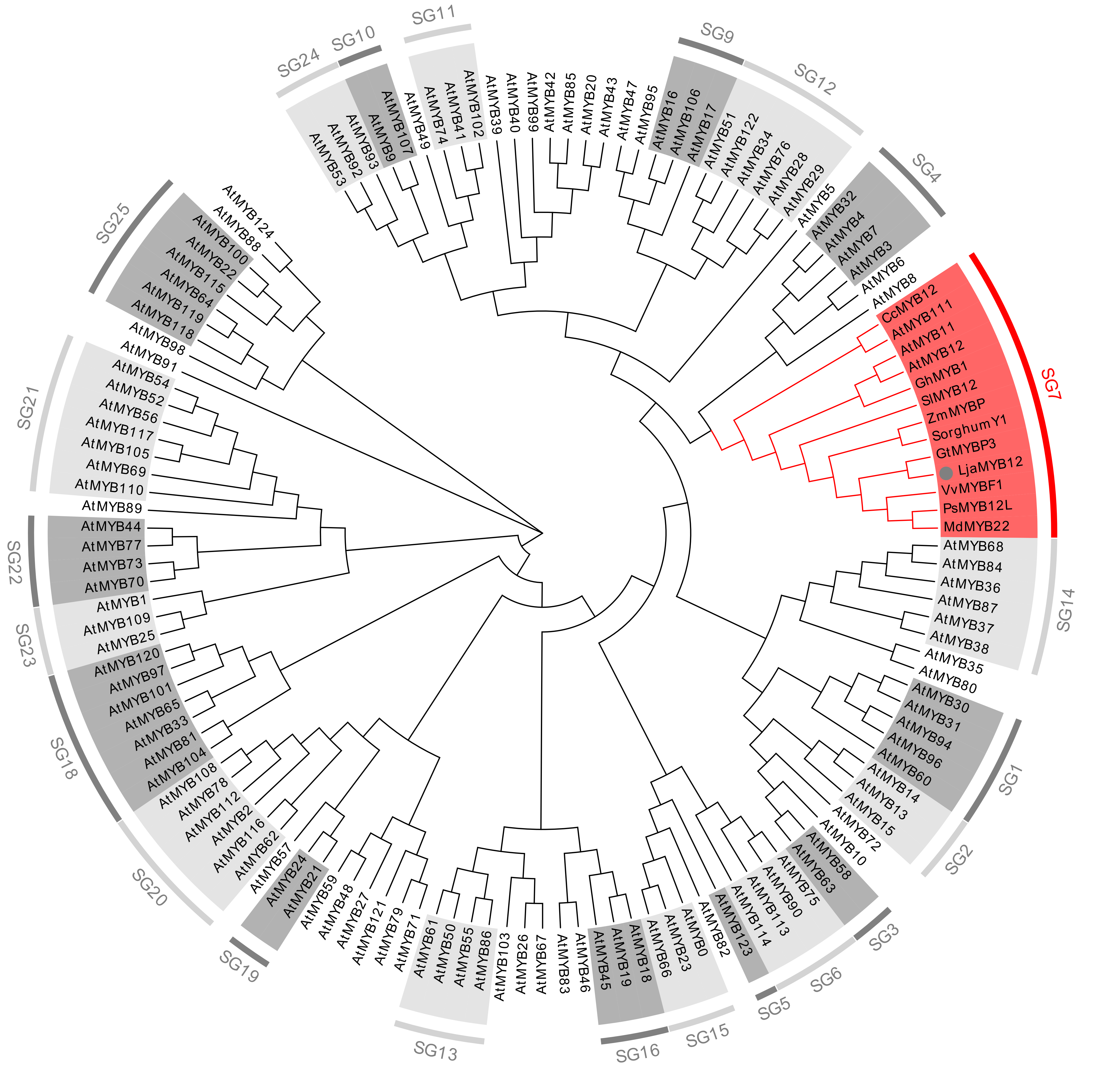

Supplementary Figure 1. Phylogenetic analysis of LjaMYB12, Arabidopsis R2R3-MYBs and representative subgroup 7 MYBs in other plants. The Arabidopsis R2R3-MYB sequences were accessed from TAIR (https://www.arabidopsis.org/). The representative subgroup 7 MYBs are CcMYB12 (AXF92691.1) from *Cynara cardunculus var. scolymus*, GhMYB1 (CAD87007.1) from *Gerbera hybrid cultivar*, GtMYBP3 (BAM71801.1) from *Gentiana triflora*, MdMYB22 (AAZ20438.1) from *Malus domestica*, PsMYB12L (QBK15080.1) from *Paeonia suffruticosa*, SlMYB12 (ACB46530.1) from *Solanum lycopersicum*, Sorghum Y1 (AAX44239.1) from *Sorghum bicolor*, VvMYBF1 (ACT88298.1) from *Vitis vinifera*, and ZmMYBP (P27898.1) from *Zea mays*.
